# A European Mitochondrial Haplotype Identified in Ancient Phoenician Remains from Carthage, North Africa

**DOI:** 10.1371/journal.pone.0155046

**Published:** 2016-05-25

**Authors:** Elizabeth A. Matisoo-Smith, Anna L. Gosling, James Boocock, Olga Kardailsky, Yara Kurumilian, Sihem Roudesli-Chebbi, Leila Badre, Jean-Paul Morel, Leïla Ladjimi Sebaï, Pierre A. Zalloua

**Affiliations:** 1 Department of Anatomy and Allan Wilson Centre, University of Otago, Dunedin, New Zealand; 2 Department of Biochemistry, University of Otago, Dunedin, New Zealand; 3 School of Medicine, Lebanese American University, Byblos, Lebanon; 4 National Heritage Institute, Tunis, Tunisia; 5 Archaeological Museum, American University of Beirut, Beirut, Lebanon; 6 Université d’Aix-Marseille, Centre Camille Jullian, Aix-en-Provence, France; IPATIMUP (Institute of Molecular Pathology and Immunology of the University of Porto), PORTUGAL

## Abstract

While Phoenician culture and trade networks had a significant impact on Western civilizations, we know little about the Phoenicians themselves. In 1994, a Punic burial crypt was discovered on Byrsa Hill, near the entry to the National Museum of Carthage in Tunisia. Inside this crypt were the remains of a young man along with a range of burial goods, all dating to the late 6^th^ century BCE. Here we describe the complete mitochondrial genome recovered from the Young Man of Byrsa and identify that he carried a rare European haplogroup, likely linking his maternal ancestry to Phoenician influenced locations somewhere on the North Mediterranean coast, the islands of the Mediterranean or the Iberian Peninsula. This result not only provides the first direct ancient DNA evidence of a Phoenician individual but the earliest evidence of a European mitochondrial haplogroup, U5b2c1, in North Africa.

## Introduction

The Phoenicians are recognized as one of the great early civilizations of the Mediterranean. First recorded as the descendants of the Canaanites, they inhabited the shores of the eastern Mediterranean and dominated the maritime trade routes of both the eastern and, later, the western Mediterranean during the second and first millennium BCE. The creators of the first alphabet, the Phoenicians documented their own records on papyrus and parchment which, unfortunately, disintegrate over time leaving behind limited historical information. The main Phoenician coastal cities, Tyre, Sidon, Byblos and Arwad, located in what is now Lebanon and southern Syria, have been continuously occupied, so rarely subjected to major archaeological excavations. As a result, we actually know little about the Phoenicians other than what was written about them by the Greeks and Egyptians [1].

At the advent of the mass migration and destruction caused by the Sea-peoples throughout the eastern Mediterranean in the later part of the 2nd millennium, the Phoenician city-states were the only ones to escape destruction, with the notable exception of Arwad, which had been under Hittite occupation [2, 3]. It was the clearing off of neighboring powers by the Sea-peoples that allowed the Phoenicians to re-assert their independence and in fact explore and move farther westward establishing a vast array of colonies throughout the Mediterranean region.

Known for their voyaging and trading skills, craftsmanship, and their access to the highly prized purple dye, from which the Greeks derived the name for these maritime traders, the Phoenicians had an important influence across the Mediterranean and beyond. While there have been claims that they had circumnavigated the African continent, there is clear evidence that they reached as far westward as the Atlantic coasts of Spain and Morocco [4]. It has also been suggested that Phoenician contact via the tin route reached as far as the south of Britain [4], though no archaeological evidence to date has been found to support this claim.

The primary strategy for Phoenician expansion and influence was the establishment of trading posts in cities across the Mediterranean where mobile Phoenician traders would interact with locals. However, they also established a few true and permanent Phoenician colonies. The most well known Phoenician settlement was the city of Carthage in Tunisia, founded by Queen Elissa (also known as Dido) and her followers who arrived from the Phoenician city of Tyre around 813 BCE. Carthage grew from a small Phoenician trading port to one of the most powerful cities in the Mediterranean. Phoenician domination was to be eventually eroded and ultimately replaced by the Greek and later the Roman might in the Mediterranean. Carthage was destroyed by the Romans in the Third Punic War (149–146 BCE), and by the end of the first millennium, the world only knew of the Phoenicians from biased Roman and Greek history books [1].

Byrsa Hill was the highest point in the city of Carthage and was the site of a Phoenician acropolis. Today it is the location of the National Museum of Carthage. In 1994, gardeners planting a tree at the front of the Museum discovered a 4 meter deep shaft which lead to a tomb holding two carved sandstone sarcophagi. One of the sarcophagi was empty, but the other contained the undisturbed skeletal remains of a young man. Funerary goods placed in the sarcophagus and above the lid of the tomb include two commercial Punic amphorae, a lamp, an ivory box of ointments, food offerings and several amulets which all date to the late sixth century BCE [5].

An osteological analysis of the young man from Byrsa, or Ariche, as he has become known, determined that he was approximately 1.7 m tall and aged between 19 and 24 years, and a craniometric analysis indicated likely Mediterranean/European ancestry as opposed to African or Asian [5, 6]. A full reconstruction of the young man was undertaken and this was part of an international exhibition organized by the National Heritage Institute of Tunis and the Carthage Museum. In 2014 the skeletal remains of the young man from Byrsa and accompanying exhibition were taken to the Archaeological Museum of the American University of Beirut where we were allowed to remove small bone samples for ancient DNA analyses to identify the origins of the young man and shed light on the genetic ancestry of the ancient Phoenicians of Carthage.

Our previous research on the genetic ancestry of the Phoenicians focused on analyses of modern Y chromosome variation in historically Phoenician and neighboring non-Phoenician sites across the Mediterranean [7]. Given that the Phoenician traders were primarily men, it was recognised that there should be remnants of the genetic “footprint” of Phoenician Y chromosomes spread across the region. Weak but systematic signatures were recognised which linked the Y chromosome STR haplotypes across Phoenician sites, with six Y-STR markers of likely Phoenician introduction identified. Here we present the first ancient DNA study of Phoenician remains and the first complete ancient Phoenician mitochondrial genome.

## Materials and Methods

### Ethics Statement

All modern Lebanese samples were collected from donors after they had given their written informed consent to the project and to the data analysis. This project was approved by the IRB of the Lebanese American University.

### Ancient DNA sampling

Two small pieces of rib were removed from the skeleton of the young man from Byrsa, under sterile conditions, and were sent to the University of Otago aDNA facility for analyses.

### DNA extraction

All DNA extraction and Illumina sequencing library preparation before PCR amplification were conducted in the purpose-built University of Otago aDNA facility [8].

The bone was treated with UVC light (λ = 254nm) for 45 minutes on each side prior to grinding with a sterile pestle and mortar. The interior cancellous portion of the rib was targeted for extraction which also reduces the chances of including surface contamination in the extraction process. Around 250 mg of bone was used for DNA extraction following the silica based extraction protocol [9]. An extraction blank was run alongside the sample.

### Preparation of Illumina Sequencing Libraries

An Illumina sequencing library was produced directly from the aDNA extract by using custom Illumina shotgun adapters [10]. The extraction blank was treated as a normal sample. To the samples, a P7 and P5 adapter were ligated to produce Illumina sequencing libraries. The number of PCR cycles to reach plateau were determined using quantitative PCR (qPCR) on a Strategene MxPro 3000P system using SYBR Green dye (ABI). Products from the qPCR were run out on 2% agarose gels and checked for the presence of adapter-ligated libraries. The libraries were then indexed and “immortalised” by PCR amplification. To reduce PCR artefacts, the amplification was stopped once plateau was reached. Immortalised libraries were purified using Qiagen MinElute purification columns following the manufacturer’s protocol.

### In-Solution Hybridisation Capture

One PCR of 1 μL immortalised library in a 50 μL reaction was performed to obtain 2 μg of Illumina sequencing library. Only 10 cycles of PCR were run at this point to avoid reaching the PCR plateau point. The library was purified and concentrated using Qiagen MinElute purification columns following the manufacturer’s protocol. Human mtDNA was enriched in the Illumina library by in-solution hybridization capture [11] with some modifications. Modern human DNA of a known European haplogroup (T2b) was used to produce the hybridization capture probe. In contrast to the published protocol [11], the library was capture-enriched individually. The enriched library was then amplified via PCR for 20 cycles, stopping well before the plateau was reached. The post-capture library was then purified using Qiagen MinElute purification columns following the manufacturer’s protocol. The resulting library was quantified using a Qubit, prior to sequencing on an Illumina MiSeq sequencing platform in a 2 x 75 base paired-end run.

### Modern DNA extraction and sequencing

The complete mitochondrial genomes for 47 modern Lebanese samples, all previously identified as belonging to mitochondrial haplogroup U, based on HVR analysis [12], were amplified in two long range PCR products, produced using primers HUM-LR1 and HUM-LR2 described previously [13]. The PCR products were pooled and 1 μg of the products, in equal molar concentrations, were mechanically sheared using sonication to produce fragments of approximately 500 bp in length. Blunt end repair, ligation of sequencing adaptors, sample barcoding and pooling were carried out following Meyer and Kircher [14] and Kircher and Kelso [15] with modifications for Illumina sequencing adaptors. Pooled samples were sequenced on one lane of the Illumina MiSeq in a 2 x 250 base paired-end run with version 2 chemistry. All members of the research team who handled the ancient Phoenician sample, both in the collection process and in the laboratory, were also sequenced to determine their mitochondrial haplotypes to assess the possibility of contamination of the ancient sample.

### Ancient DNA processing and analysis

Raw reads from the ancient Phoenician were processed to remove sequencing adaptors and merge paired end fragments, which overlapped by at least 11 base-pairs, using AdapterRemoval (v1.5.4) [16]. Reads were also processed to remove stretches of Ns, bases that had a low quality score (<30), and short reads (<25). To reduce the impact of contamination due to laboratory reagents, all read mapping was done onto a composite reference genome consisting of the Cambridge Reference Sequence for humans (*Homo sapiens*, GenBank NC_012920), in addition to mitochondrial reference genomes from cow (*Bos taurus*, GenBank NC_006853), pig (*Sus scrofa*, NC_0012095.1), and chicken (*Gallus gallus*, GenBank NC_001323.1). The contamination ratio was determined by calculating the ratio of all reads with mapping quality > = 20 for each reference, compared with those for the human mitochondrial reference sequence [17].

Ancient DNA is usually characterised by C→T transitions at the 5' ends of the molecule, and following double stranded library preparation G→A transitions at the 3' ends of the molecules [18]. Reads were aligned to the composite reference genome using BWA (0.7.10) [19] with recommended ancient DNA settings [20]; specifically, seeding was disabled (-l 1024), the number of gap opens was set to 2 (-o 2), and the maximum edit distance was set to 0.03 (-n 0.03). PCR duplicates were removed from the merged reads using a python script originally developed for the Neanderthal genome project [21]. These PCR duplicates were also removed from the unmerged reads using Picard’s MarkDuplicates tool (http://broadinstitute.github.io/picard/). To confirm the authenticity of our ancient DNA, the program mapDamage (v2.0.2–9) [22] which identifies characteristic aDNA damage patterns, was used with the '-rescale' option to lower the quality score of likely damaged sites. The plots of characteristic damage patterns were individually created for merged and unmerged reads. Finally, Ts (thymines) found at the 5' end of a read and Gs (guanines) at the 3' end of the read, within the first two bases, had their quality scores rescaled to zero. The presence of modern human DNA in the sequencing libraries presents a challenge for ancient DNA, as it can introduce major biases into downstream population genetic and phylogenetic [23, 24]. Modern human mtDNA contamination levels were estimated using the ContamMix package [25].

Reads that mapped to the human reference genome were extracted from the PMD filtered BAM file and a variant call file (VCFs) was generated using the GATK Haplotype Caller (v3.3) [26] with settings specific for haploid genomes, such as the mitochondrial genome. This file was filtered for mapping quality (<20), and bases supported by fewer than three reads [27, 28]. This approach was justified under the assumption that the above quality control (QC) procedure preferentially reduces the quality of the damaged positions in the sequencing reads, which would lead to a reduction in the probability that the damaged position would be called downstream. A coverage plot and a fragment length plot were created for the Phoenician samples using the ggplot2 package for the R programming language [29]. The fragment length plot was created only using the merged reads, this was done because estimation of the fragment length is error prone using unmerged reads. A consensus sequence containing indels (insertions and/or deletions) was created, and has been deposited in GenBank (KT760574). In addition to the aforementioned QC measures, non-variant sites that were supported by fewer than three reads were changed to 'Ns'. To assign a haplogroup to the Phoenician, we used HaploGrep [30] (http://haplogrep.uibk.ac.at/) for Phylotree 16 [31]. All Illumina reads generated for the ancient Phoenician have been submitted to the NCBI short read archive as sample MS10148, accession number PRJNA295854.

### Modern sequence processing and analysis

The raw reads from the modern Lebanese samples were mapped to the human mitochondrial reference genome (*Homo sapiens*, GenBank NC_012920) using the BWA [19] (v 0.7.10) maximal exact matches (MEM) command [32]. This was followed by PCR duplicate removal using Picard’s MarkDuplicates command. Haplogroups were assigned by first generating a joint-called VCF using the GATK HaplotypeCaller, converting the VCF into the HaploGrep input format, and finally assigning a haplogroup to each Lebanese sample with HaploGrep [30] (http://haplogrep.uibk.ac.at/). All 47 complete modern Lebanese mitochondrial genome sequences have been submitted to GenBank (accession numbers KT779157-KT779203).

### Phylogenetic Analysis

To investigate the phylogenetic relationship of the ancient Phoenician (KT760574) to other U5b2c sequences, we obtained the publicly available U5b2c sequences from GenBank and the supplementary information of a recent publication investigating present day mitochondrial diversity in Portugal [33]. A maximum likelihood (ML) tree was generated with MEGA using the HKY + I model. Preliminary analyses revealed that the sequence EF758625 and KU587510 had many missing positions reducing the phylogenetic resolution, thus these samples were removed from the final analysis.

## Results

### The complete ancient mtDNA genome

**Fig 1:**
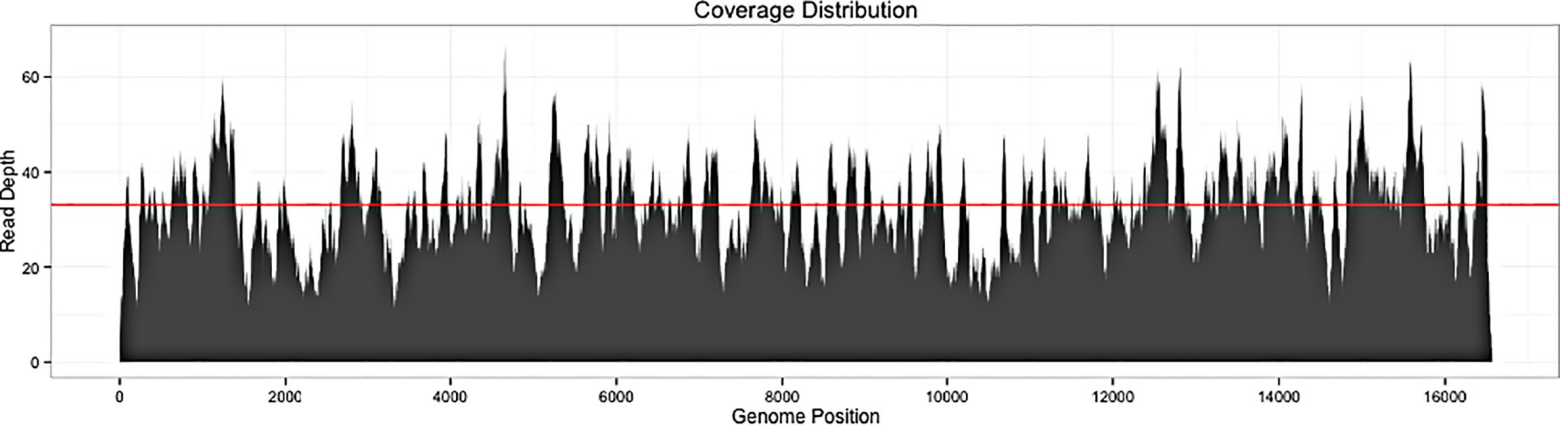
Final read depth and coverage for the mitochondrial genome of the ancient Phoenician from Carthage. The red line shows the mean read-depth of 33.15x.

A total of 11,129,232 paired-end reads were returned for the ancient Phoenician. After bioinformatics processing of these raw reads, which included adaptor removal, PCR duplicate removal, read merging, and quality filtering, 4,851 reads remained which mapped uniquely (MAPQ > 20) to the human mitochondrial reference genome. This represents an endogenous mitochondrial DNA fraction of 0.0004%. The final read-depth for the ancient Phoenician was 33.1x (Fig 1). Analysis of the variable sites with the online tool Haplogrep revealed that the ancient Phoenician had the mitochondrial haplotype U5b2c1.

### Sequence authenticity

**Fig 2:**
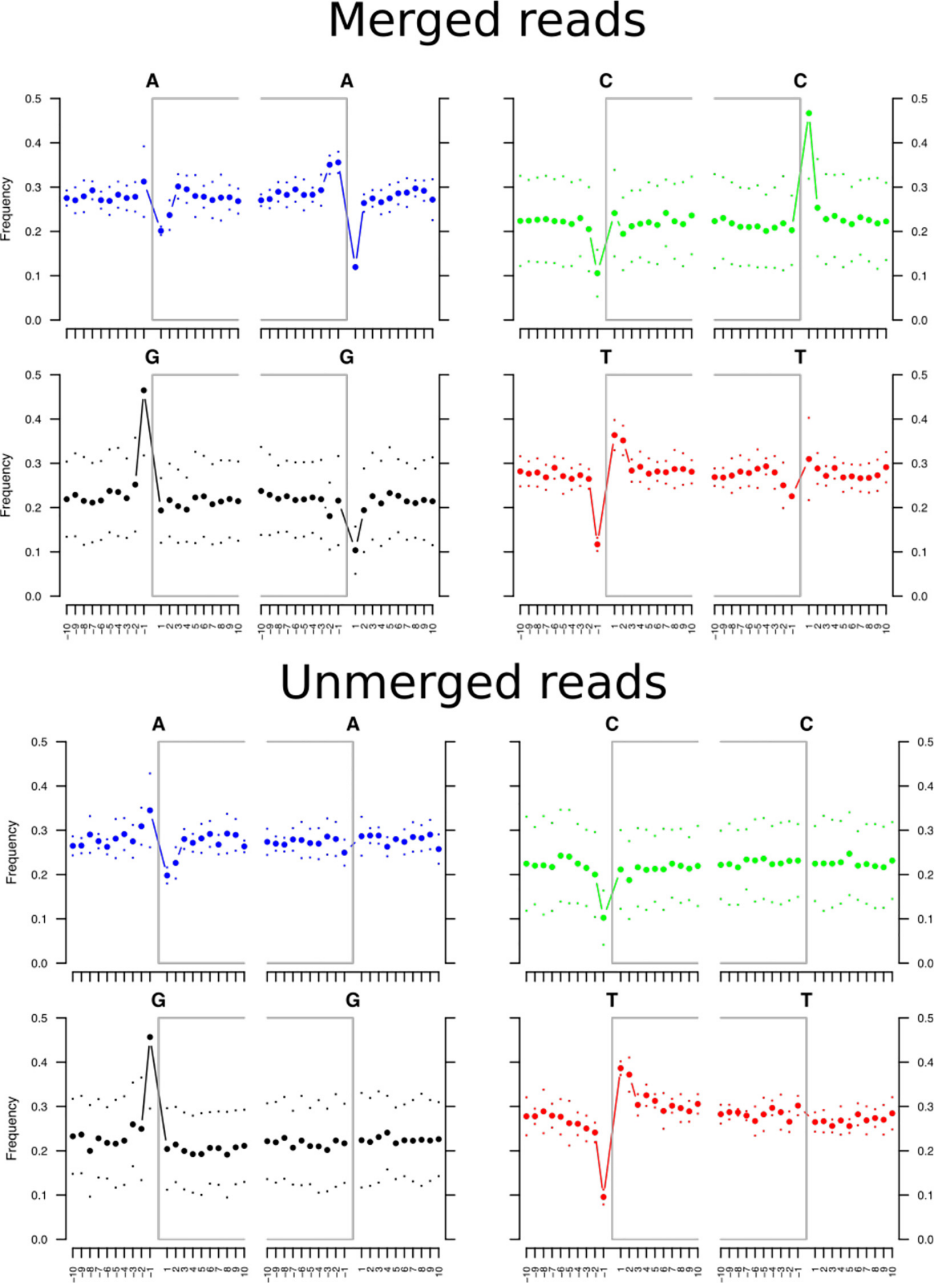
Base frequency 5’ and 3’ of strand breaks. The gray brackets indicate the start and end of molecules (strand breaks). Frequencies are displayed for A, G, C, and T for the 10 bases 5’ and 3’ of the breaking site. The top panel shows that merged reads have an elevated frequency of purines (A and G) before strand breaks; this is consistent with the DNA molecules being ancient [36]. The bottom panel shows that unmerged reads have an elevated frequency of purines (A and G) before 5’ strand breaks but not 3’ strand breaks, this has occurred because mapDamage utilizes only the first read of a pair to calculate these frequencies. As the end of the first-read does not represent the end of the molecule, we would not expect to see an elevated purine frequency at the 3’ breaking site. Therefore, these results are consistent with the longer unmerged reads being ancient.

**Fig 3:**
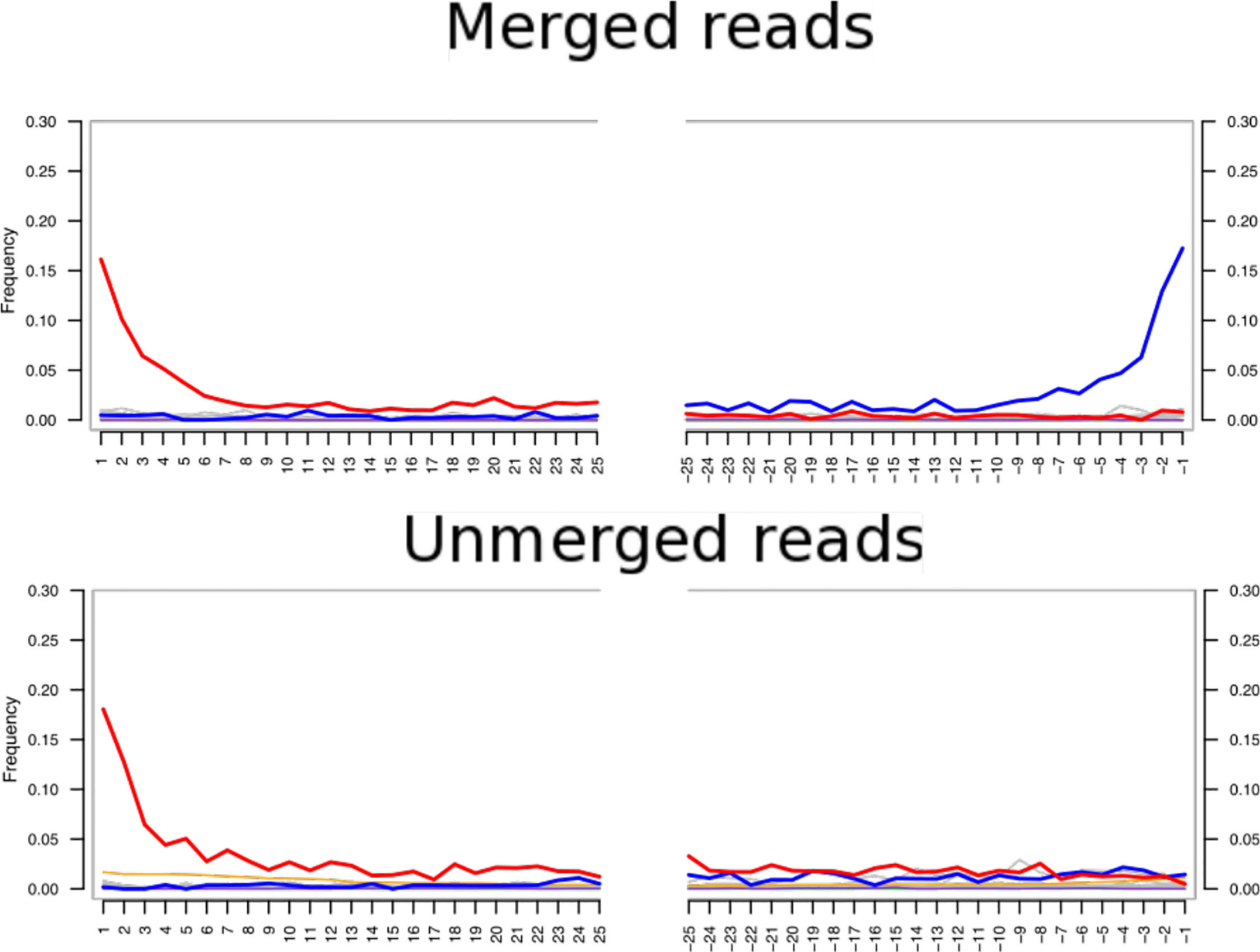
C to T nucleotide misincorporations for the first and last 25 bases of endogenous mtDNA fragments. Red T; green C; blue A; purple G. The top panel shows that merged reads have an increased frequency of T at the 5’ end and A at the 3’ end (G to A misincorporation on the opposite strand of C to T misincorporation), a typical pattern of ancient DNA damage [35, 36]. The bottom panel shows that unmerged paired-end reads have an elevated frequency of T at the 5’ end, but in contrast with the above panel no increase in A at the 3’ end. This has occurred because mapDamage utilizes only the first read of a pair to calculate these frequencies. As the end of the first-read does not contain the sequence of the entire molecule, we would not expect to see an elevated A frequency at the end of the read. Therefore, these results are consistent with the unmerged reads originating from larger DNA fragments being ancient.

For human ancient DNA studies two potential sources of contamination exist, these are the reagents [34] and modern human DNA [35]. To reduce the impact and to estimate the effect of reagent contamination, we performed all read mapping to a composite mitochondrial reference genome that included the major reagent contaminators cow, chicken, and pig [34]. Only 4 reads mapped confidently to these genomes. To assess the totality of modern human DNA contamination we used the program ContamMix with a set of 311 representative modern human mtDNA sequences. This estimated that the ancient Phoenician had been contaminated with 1.1% (95% C.I. 0.004%-2.34%) of modern human DNA. The deamination patterns and fragment lengths (Figs 2 and 3) were consistent with those expected from ancient DNA [36]. For example, the observed C to T misincorporation rate for the first base of the 5’ end was 0.16. This is outside the maximum rate of 0.05 for modern samples (<117 years old) and within the range of what is to be expected for a sample that dates to the 6^th^ Century BCE (see Fig 2 in [36]).

### Haplogroup assignment and phylogenetic analysis

**Fig 4:**
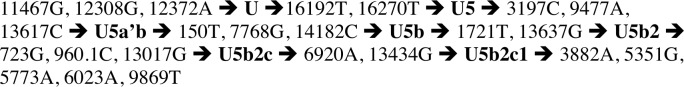
Key variable sites from rCRS found in the young man from Byrsa, with the marker path of mutations defining Haplogroup U to U5b2c1. Note, the mutation 16192T was not present in our sample.

The complete mitochondrial genome of the young man from Byrsa was identified as belonging to haplogroup U5b2c1 possessing all of the defining mutations with the exception of 16192T, one of the two mutations defining U5, which has shown to be unstable with frequent reversions [37]. He also had five additional, coding region mutations (Fig 4). None of the researchers who collected or processed the Phoenician sample have mitochondrial haplotypes belonging to Haplogroup U. All methods to avoid contamination were applied and the possibility of modern contamination in the sequence obtained was assessed using standard aDNA authenticity criteria and shown to be unlikely, thus we are confident that the haplotype identified is indeed that of the young man from Byrsa.

**Fig 5:**
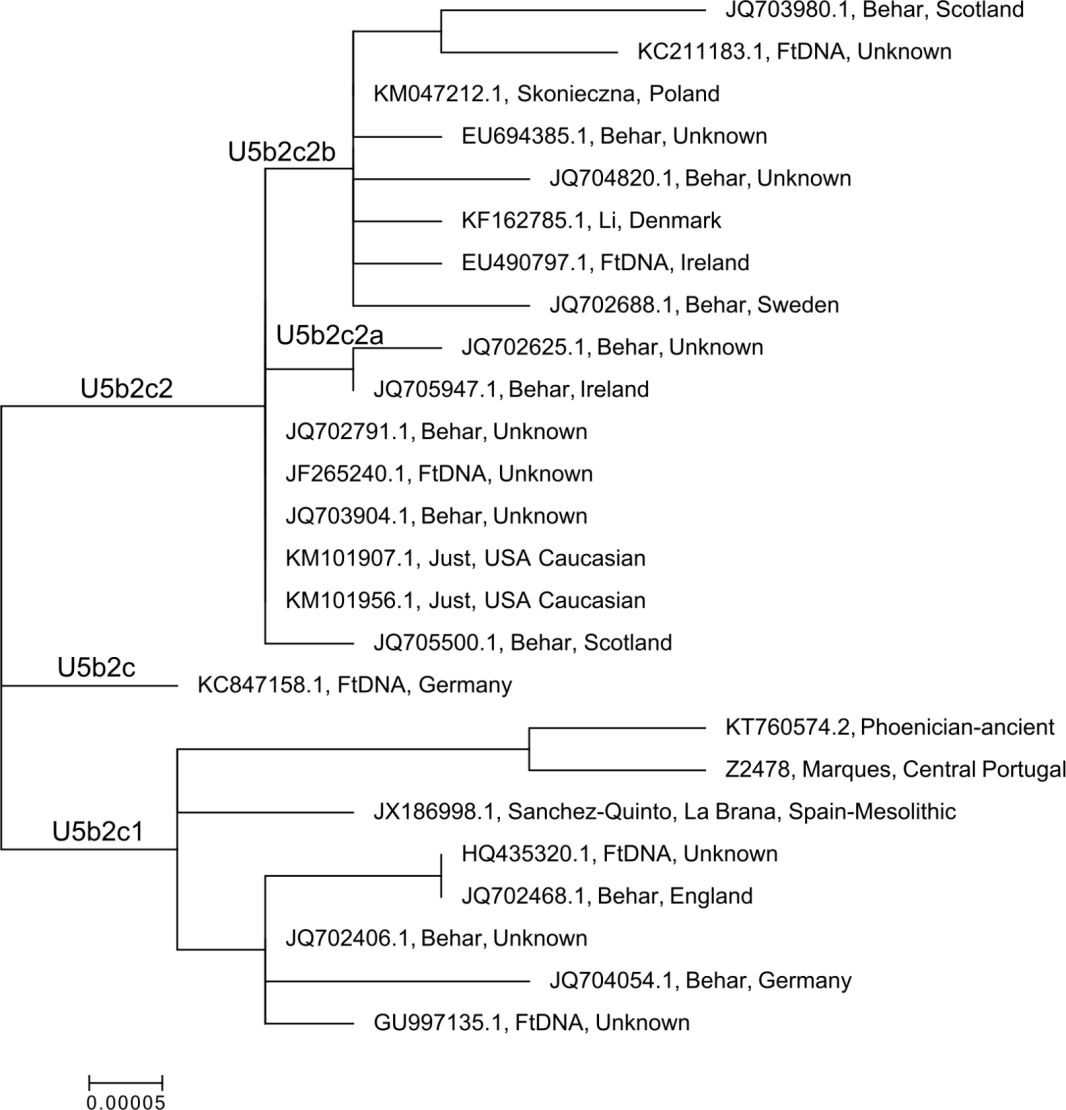
A maximum likelihood (ML) tree for the ancient Phoenician (KT760574) and other publicly available U5b2c sequences. All samples other than our Phoenician and the La Braña sample are from modern populations. Each node is annotated with the GenBank accession number or sample identification (e.g. Z2478 from [33]), source or author if published, and the origin of the sample if recorded [33, 38–42].

A phylogenetic analysis of all U5b2c sequences available in GenBank or from published supplementary data (Z2478) is shown in Fig 5.

### Modern mitochondrial genome sequencing

Given the reputed Lebanese origins of the founders of Carthage, we undertook full mitochondrial genome sequencing of 47 modern Lebanese samples that had previously been typed to Haplogroup U through analysis of the HVR-1 [12]. Haplogroups identified and haplogroup frequency are shown in Table 1. Only seven of the modern Lebanese samples belonged to Haplogroup U5 and of those, two were U5b, but neither belonged to U5b2c or derived haplotypes.

**Table 1:** Mitochondrial haplogroups identified via complete mitochondrial genome sequencing in 47 modern Lebanese previously identified (based on HVR-1) as belonging to Haplogroup U.

Haplogroup	N
U1a1	1
U1a1a	3
U1b	4
U2d2	2
U2e1'2'3	1
U3a	4
U3a1	1
U3b	7
U3b1	2
U3b2a	4
U3b2a1	2
U3b3	2
U3c	1
U4b3	2
U5a1a1g	2
U5a1g	2
U5a2b	1
U5b2b	1
U5b3a2	1
U6a7a1b	1
U6b1a	1
U7b	1
U8a1a	1
**Total**	**47**

## Discussion and Conclusion

Haplogroup U5 is considered to be one of the most ancient haplogroups in Europe and is believed to have arisen there [43]. The coalescence time estimate for U5 is 29.6 kya (22.7–37.2 kya) [25] and 20–24 kya for U5b [44]. It is not uncommon in Mesolithic European populations, particularly those from Central and Eastern Europe [45]. Haplogroup U5b2c1 has been identified in both La Braña 1 and 2, the 7000 year-old remains recovered from the La Braña-Arintero site in León in Northwestern Spain [42]. Our Phoenician differed from the La Braña 1 complete mitochondrial genome at eight sites (positions 3882, 5351, 5773, 6023, 9869, 16069, 16126, and 16192). The mutations at sites 16069 and 16126 appear to be private mutations for La Braña.

It appears that the U5b2c1 haplogroup is rare in modern populations, with only a few modern sequences published [33, 38, 46] or available in public databases (Family Tree DNA). All of the reported U5b2c1 carriers are of presumably (if not specifically stated) European ancestry, from Spain, Portugal, England, Ireland, Scotland, the United States and Germany. Three of the additional non-defining mutations found in our Phoenician, 5351G, 6023A, and 9869T, are shared with one “European” sample [46] and an individual from central Portugal [33]. Interestingly, Fig 5 shows that our Phoenician sample is most closely related to the modern sample from central Portugal. The unidentified “European” sequence (EF758625) which was deleted from the analysis due to missing data, also carries the three mutations which define the branch on which we find the Phoenician and the Portuguese sample. A separate branch within U5b2c1 contains five samples, from England and Germany or otherwise unidentified as to location of origin, with the La Braña Mesolithic sample (JX186998) on its own branch. Given the limited numbers of published full mitochondrial genomes, it is difficult to identify a likely origin for the mutations defining the Phoenician and Portuguese branch, but it is currently not inconsistent with a Southwest European origin. While the U5b2c2 sequences all originate in Central- and North-Western European or likely derived populations (e.g. US Caucasian), and the U5b2c sequences in GenBank (KC847158 and KU587510, which was excluded from the analysis due to missing data) originate in Germany and England respectively (both samples from Family Tree DNA), it is likely that U5b2c originated in Central/Northwestern Europe. Future complete mitochondrial genome sequencing of European populations, both ancient and modern, will undoubtedly provide more information regarding the distribution and frequency of the U5b2c1 haplogroup across the continent and thus will help us to reconstruct its evolutionary history.

It is generally argued that Mesolithic populations carrying high frequencies of U5 were replaced by Neolithic populations expanding into Western Europe during the Neolithic transition [47]. Recent analyses of ancient DNA suggest that there were two waves of Neolithic expansions that dramatically influenced the genetic makeup of Western Europe [48–51]. An early Neolithic expansion out of Anatolia, occurring some 7000–9000 years ago, moved into Western Europe and replaced many Mesolithic Hunter-Gatherer populations, resulting in an increase of Near Eastern mitochondrial lineages. After this initial invasion of Neolithic farmers, there appears to have been a resurgence in the Western Hunter-Gatherer genetic signatures, followed by a second migration and replacement in many locations from the Eurasian Steppes, associated with the spread of the Yamnaya culture, beginning some 4500 years ago [49]. Several other more regionally focused aDNA studies, however, have shown that there are different patterns of population replacements in various regions of Europe [52, 53]. Unlike the early Neolithic arrival in the Iberian peninsula, which likely arrived in coastal regions [54, 55], it is possible that the second, Eurasian Steppe derived, replacement occurred later or had less of an impact on the southern and western Iberian coast and other Mediterranean coastal regions than in more northern or eastern inland locations [49, 53] and thus the Western Hunter-Gatherer mtDNA lineages, including U5b2c1, may have been more common there at the time of Phoenician contact and settlement.

While the U5b frequency in modern populations in Western Europe is less than 2% it is much rarer in today’s Near Eastern populations [12]. In our full mitogenome sequencing results of 47 modern Lebanese U samples, we found only two individuals who carried haplotypes belonging to U5b2 and neither of these belonged to the U5b2c1 subgroup. It has been demonstrated, however, that modern Levantine populations do not reliably represent the haplogroups present in the Neolithic period, though haplogroup U5 was only found at low frequencies in pre-pottery Neolithic (PPNB) samples from the Levant [54]. U5b is found in only 0.4% of the modern populations from the Iberian peninsula, and only 0.18% Europe-wide [42], which provides us with further confidence of the authenticity of our ancient DNA result. Only the Saami, in northern Scandinavia, retain high levels of U5, and U5b1b in particular, with frequencies over 50% in some Saami populations [56].

Interestingly, haplogroup U5 and U5b have been identified in modern populations from North and Northwest Africa [57–59]. It must be noted, however, that there are few North African complete mitogenomes publicly available and HVR sequencing alone cannot identify beyond the U5b haplogroup (with the C150T HVR mutation) since the seven derived defining mutations (C1721T and A13637G, which define U5b2; A723G, 960XC and A13017G, which define U5b2c; and C6920a and A13484G, which define U5b2c1) are all in the coding regions. Achilli, et al. [59], using full mitochondrial genome sequencing identified a U5b1b1 cluster that grouped Amazigh (North African Berbers) and Saami populations. This cluster is based on the control region motif (16270–150) which is present at low frequencies in Amazigh, North African and nearly all European populations with the exception of the Scandinavian Saami where it is at about 48%. The divergence time of this cluster is around 8600 years ago (+/- 2400) consistent with an expansion from Franco-Cantabrian refuge which is believed to have been a major refuge for the European hunter-gatherers prior to their post LGM expansion [44]. It is suggested that European haplogroup U5 and the more prevalent U6 “Berber cluster” diverged from a common ancestor in the Near East and spread along the north and south coasts, respectively, of the Mediterranean, as far as Iberia to the north and Cyrenaica to the south [60]. It is very plausible that descendants of the Mesolithic hunter-gatherers carried U5b1b1 and sister lineages across the Straits of Gibraltar into North Africa [59], but there is no indication of when this migration may have happened. While it is possible that U5b2 haplogroups were also carried across the Straits of Gibraltar prior to Phoenician arrival in North Africa, our result now provides a minimum date of arrival. We can now say that U5b2c1 was present by the late 6^th^ century BCE.

Though it is believed that Carthage was established by colonisers from Tyre, in what is today Lebanon, it is unlikely that our Phoenician young man had maternal ancestry that traced back to this founding population as the haplogroup U5b2c has not been identified in our modern Lebanese samples or in ancient (early Neolithic, PPNB) remains from the Levant [54]. Given that haplogroup U5b2c1 has not been previously reported in North Africa, we suggest that the ancestry of our young man is likely traced to some population across the Mediterranean, which is consistent with his proposed European cranial traits [6].

So how did a young Phoenician man, with a European mitochondrial lineage end up in Carthage, North Africa? The earliest Phoenician site in Iberia is believed to be Gadir or Cadiz as it is known today, established in 1110 BCE [1]. Phoenician colonies were also established in Ibiza, southern Sardinia, western Sicily and along the southern shores of the Iberian Peninsula [1, 61, 62] and, later, these were also major Carthagenian trade ports. It has been suggested that the Phoenician east-west trade route from Tyre and Sidon across the Mediterranean and through the Straits of Gibraltar departed following a northerly route, travelling with the prevailing winds and tides westwards, stopping in Cyprus, Sicily, Ibiza and several locations along the southern coast of Spain, reaching Cadiz and beyond. Return trips back to the Levant took a southerly Mediterranean route, again, with the prevailing currents, sailing along the North African coast [1]. While early Phoenician occupation of and trade in the western Mediterranean would have been from the Levant, Carthage, once established, came to dominate the western Mediterranean trade networks, but would most likely have continued with this well travelled, counterclockwise circular route.

To date, as far as we can determine, U5b2c1 has not been identified in the Balearic Islands, Sardinia, or Sicily [62–64], though it may have been on these islands in the past and lost due to drift. While some of the reported modern individuals carrying the U5b2c1 haplogroup today are found in the Iberian peninsula, they are also found in Britain, Germany, the United States, and undefined “Europe”, so we cannot say with certainty that the haplogroup is more than European. However, the highest frequency of the haplogroup U5b today is in the Iberian peninsula and U5b2c1 was also present there in Mesolithic hunter gatherers [42]. While many of the Mesolithic populations carrying these ancient U5b European lineages were replaced by later Neolithic peoples, perhaps some of these lineages persisted longer in the extreme southern part of the Iberian peninsula, on the offshore islands, or other island or coastal locations of Phoenician influence and individuals carrying these ancient European lineages were transported to Carthage via the Phoenician/Punic trade networks.

We know of no other U5b2c haplotypes recorded in North Africa [58]. Our Phoenician young man significantly predates the Moorish expulsion from Iberia (1492 AD) which resulted in the Andalusian communities that were established in Tunisia. Thus we now have a minimum date for the introduction of the European derived U5b2c1 haplotype to North Africa and its direct association with a Phoenician individual. Further work on aDNA from Phoenician remains from throughout the Mediterranean, including the homeland region in Lebanon is on-going. This research will help us better understand the origins and impact of Phoenician peoples and their culture throughout the Mediterranean region and beyond and better reconstruct ancient population migrations and trade and exchange networks and the degree to which these influenced genetic variation seen in the Mediterranean region today.
